# Factors associated with the use of adjuvant radiation therapy in stage III melanoma

**DOI:** 10.3389/fonc.2023.1005930

**Published:** 2023-02-01

**Authors:** Amber L. O. King, Victor Lee, Beverly Yu, Fatima N. Mirza, Cheryl K. Zogg, Daniel X. Yang, Thuy Tran, Jonathan Leventhal, Yi An

**Affiliations:** ^1^ Department of Dermatology, Yale School of Medicine, New Haven, CT, United States; ^2^ Department of Therapeutic Radiology, Yale School of Medicine, New Haven, CT, United States; ^3^ Department of Surgery, Yale School of Medicine, New Haven, CT, United States; ^4^ Department of Medicine (Medical Oncology), Yale School of Medicine, New Haven, CT, United States

**Keywords:** adjuvant radiation therapy, melanoma, radiation oncology, skin neoplasm, trends, NCDB

## Abstract

**Objective:**

The role of radiation therapy (RT) in melanoma has historically been limited to palliative care, with surgery as the primary treatment modality. However, adjuvant RT can be a powerful tool in certain cases and its application in melanoma has been increasingly explored in recent years. The aim of this study is to explore national patterns of care and associations surrounding the use of adjuvant RT for stage III melanoma.

**Methods:**

The National Cancer Data Base (NCDB) was used to identify patients who were diagnosed with stage III melanoma between 2004 and 2014. Exclusion criteria included those with distant metastatic disease, *in-situ* histology, no confirmed positive nodes, palliative intent therapy, and dosing regimens inconsistent with National Comprehensive Cancer Network (NCCN) guidelines for adjuvant RT in melanoma. Patients treated with and without adjuvant RT were compared and factors associated with use of adjuvant RT were identified using multivariable logistic regression analyses.

**Results:**

A total of 7,758 cases of stage III melanoma were analyzed, of which 11.7% received adjuvant RT. The mean age of the overall cohort was 58.5 years, and the majority of patients were male (64.7%), white (96.6%), on private insurance (51.3%), and presented to a non-high-volume facility (90.3%). Multivariable regression analyses revealed that patients who present to the hospital in 2009-2014 as compared to 2004-2008 (odds ratio [OR] 1.61, 95% confidence interval [CI] 1.36-1.92), had 4 or more positive nodes (OR 4.30, 95% CI 3.67-5.04), and had microscopic residual tumor (OR 2.11, 95% CI 1.46-3.04) were more likely to receive adjuvant RT. Factors that were negatively associated with receiving adjuvant RT included female gender (OR 0.72, 95% CI 0.61-0.85) and median income of at least $63,000 (OR 0.66, 95% CI 0.52-0.83).

**Conclusions:**

This study demonstrates the rising use of RT for stage III melanoma in recent years and identifies demographic, social, clinical, and hospital-specific factors associated with patients receiving adjuvant RT. Further investigation is needed to explore disease benefits to improve guidance on the utilization of RT in melanoma.

## 1 Introduction

Melanoma has historically been thought to be radio-resistant, and postoperative radiation therapy (RT) has typically been sparingly used. Although the role of RT in melanoma remains controversial, RT has been established as a palliative treatment option for unresectable melanoma, and the ANZMTG 01.02/TROG 02.01 trial observed that RT following lymphadenectomy for selected patients with node-positive melanoma may reduce risk of locoregional recurrence (1).

Although surgery is the mainstay of treatment for melanoma, multiple factors affect the efficacy and eligibility for surgery. Depending on the site of malignancy, resection with completely negative margins may be difficult, if not impossible, to achieve. Comorbidities, unresectable disease, and patient preference are other possible reasons for consideration of other treatment modalities for melanoma. In these cases in which an operative approach is not recommended or not possible, RT as definitive therapy can be considered (2). Several investigators have additionally examined the impact of adjuvant RT on survival and locoregional control in melanoma (3–5). According to the National Comprehensive Cancer Network (NCCN) 2023 Guidelines, adjuvant RT can be considered for patients with high-risk desmoplastic melanoma, those at high risk for regional recurrence after resection of the primary tumor, and in cases with brain metastases (2). Despite some evidence of benefit, there is a lack of clinical framework to guide the utilization of RT in stage III melanoma and optimal dosing regimens are not well-established. No study to our knowledge has examined the factors associated with the use of adjuvant RT in melanoma overall. To this end, our study seeks to examine the trends of adjuvant RT utilization over time, and to identify demographic, social, clinical, and hospital-specific factors associated with receipt of adjuvant RT.

The aim of this study is to explore the national patterns of care regarding utilization of adjuvant RT for stage III melanoma.

## 2 Methods

### 2.1 Data source

The National Cancer Data Base (NCDB) is a joint project by the American Cancer Society and the American College of Surgeons Commission on Cancer (CoC) that includes about 70% of the newly diagnosed cases of cancer in the United States from about 1,500 hospitals with CoC-accredited cancer programs. All of the data included in the NCDB is compliant with the privacy requirements of the Health Insurance Portability and Accountability Act (HIPAA). Institutional review board approval was not needed for this study since no patient, provider, or hospital identifiers were examined.

### 2.2 Study population

**Figure 1:**
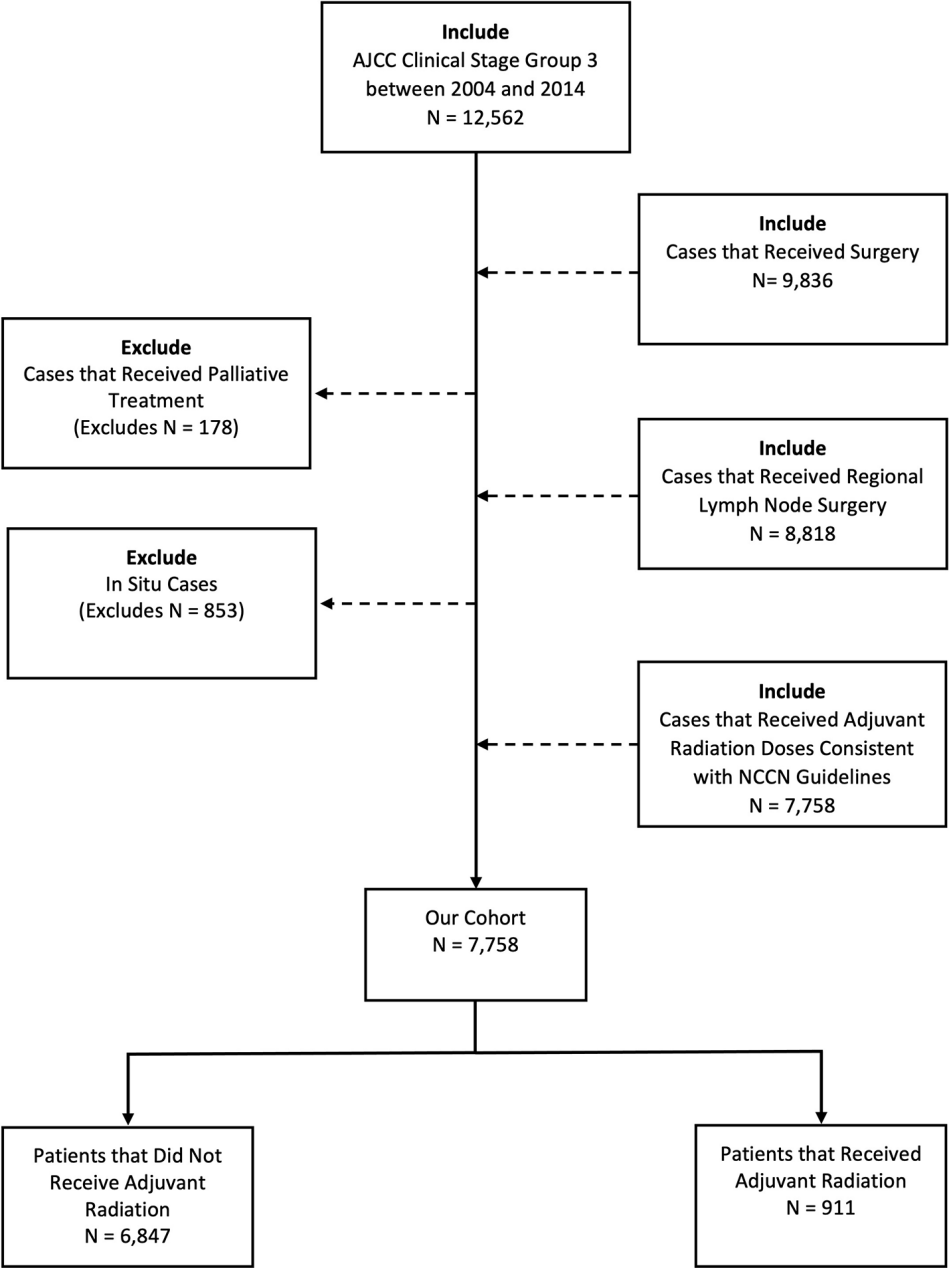
Consort diagram for inclusion criteria of the study.

The NCDB was queried for patients diagnosed with stage III melanoma between 2004 and 2014. These years were chosen around the ANZMTG 01.02/TROG 02.01 trial and were further subcategorized into two groups, 2004-2008 and 2009-2014, to achieve an approximately even bifurcation. Our inclusion criteria included patient age older than 18 years and clinical stage III disease. Patients with distant metastatic disease, *in-situ* histology, no confirmed positive nodes, palliative intent therapy, and dosing regimens inconsistent with National Comprehensive Cancer Network (NCCN) guidelines for adjuvant RT in melanoma were excluded (**Figure 1**). Potential dosing regimens of adjuvant RT per NCCN guidelines include 30 Gy in 5 fractions over 2 weeks, 48 Gy in 20 fractions over 4 weeks, and 60-66 Gy in 30-33 fractions over 6-7 weeks (2). As such, cases with adjuvant radiation doses >66 Gy were excluded.

### 2.3 Statistical analysis

Baseline differences in demographic, socioeconomic, and hospital characteristics between patients treated with and without adjuvant radiation were assessed by chi-square and ANOVA testing. Statistical significance for these analyses was set at p<0.001, and hypothesis testing was two-sided. Multivariable logistic regression analyses were performed to identify factors associated with the use of adjuvant radiation. Demographic and clinical factors that were found to be significant on univariate analysis were included in the multivariable model. Age category, high facility volume status (>90^th^ percentile volume), and Charlson-Deyo Score were also included in the multivariable model. These demographic, socioeconomic, clinical, and hospital-related factors were all included in the multivariable models. Stata Version 13.1 (StataCorp LP, College Station, TX) was used to perform data analyses.

## 3 Results

From 2004-2014, there were a total of 7,758 cases of stage III melanoma in the NCDB, of which 911 (11.7%) received adjuvant RT. Baseline characteristics of patients that did and did not receive adjuvant RT are outlined in **Table 1**. The mean age of the overall cohort was 58.5 years. The majority of patients presented in 2009-2014 (66.3%), were male (64.7%), white (96.6%), used private insurance (51.3%), and went to a non-high-volume facility (90.3%).

**Table 1:** Demographic characteristics of patients in cohort.

Predictor Variable	No Adjuvant RT (Number of Patients [% of Patients Receiving No Adjuvant RT])	Adjuvant RT (Number of Patients [% of Patients Receiving Adjuvant RT])	Total (Number of Patients [% of All Patients])	P value
Age (mean, years)	58.19	60.49	58.46	**<0.001**
Year Category				**<0.001**
2004-2008	2,380 (34.76)	232 (25.47)	2,612 (33.67)	
2009-2014	4,467 (65.24)	679 (74.53)	5,146 (66.33)	
Age Category				**<0.001**
18-49	2,026 (29.59)	198 (21.73)	2,224 (28.67)	
50-64	2,271 (33.17)	338 (37.10)	2,609 (33.63)	
65+	2,550 (37.24)	375 (41.16)	2,925 (37.70)	
Sex				**<0.001**
Male	4,358 (63.65)	660 (72.45)	5,018 (64.68)	
Female	2,489 (36.35)	251 (27.55)	2,740 (35.32)	
Race				**<0.001**
White	6,636 (96.92)	860 (94.40)	7,496 (96.62)	
Black	79 (1.15)	22 (2.41)	101 (1.30)	
Other	132 (1.93)	29 (3.18)	161 (2.08)	
Median Income Quartiles 2008-2012				**<0.001**
< $38,000	914 (13.52)	156 (17.41)	1,070 (13.98)	
$38,000 - $47,999	1,563 (23.13)	218 (24.33)	1,781 (23.27)	
$48,000 - $62,999	1,815 (26.86)	258 (28.79)	2,073 (27.08)	
$63,000 +	2,466 (36.49)	264 (29.46)	2,730 (35.67)	
Primary Payor				0.003
Not insured	349 (5.10)	45 (4.94)	394 (5.08)	
Private Insurance	3,556 (51.94)	424 (46.54)	3,980 (51.30)	
Medicaid	368 (5.37)	63 (6.92)	431 (5.56)	
Medicare	2,391 (34.92)	341 (37.43)	2,732 (35.22)	
Other Government*	97 (1.42)	24 (2.63)	121 (1.56)	
Unknown	86 (1.26)	14 (1.54)	100 (1.29)	
Facility Type**				0.990
Community Cancer Program	419 (7.08)	58 (7.04)	477 (7.07)	
Comprehensive Community Cancer Program	2,078 (35.09)	294 (35.68)	2,372 (35.16)	
Academic/Research Program	2,906 (49.07)	400 (48.54)	3,306 (49.01)	
Integrated Network Cancer Program	519 (8.76)	72 (8.74)	591 (8.76)	
Facility Location				0.211
New England	332 (5.61)	46 (5.58)	378 (5.60)	
Middle Atlantic	959 (16.19)	109 (13.23)	1,068 (15.83)	
South Atlantic	1,348 (22.76)	210 (25.49)	1,558 (23.10)	
East North Central	997 (16.84)	126 (15.29)	1,123 (16.65)	
East South Central	439 (7.41)	71 (8.62)	510 (7.56)	
West North Central	376 (6.35)	61 (7.40)	437 (6.48)	
West South Central	376 (6.35)	53 (6.43)	429 (6.36)	
Mountain	325 (5.49)	49 (5.95)	374 (5.54)	
Pacific	770 (13.00)	99 (12.01)	869 (12.88)	
High Volume Facility***				0.079
High Volume Facility	682 (9.96)	74 (8.12)	756 (9.74)	
Non High Volume Facility	6,165 (90.04)	837 (91.88)	7,002 (90.26)	
Total	6,847	911	7,758	

* The Primary Payor “Other Government” category is one of the insurance status categories listed in the NCDB Data Dictionary and refers to non-Medicaid and non-Medicare government programs, including the Veterans Health Administration and Indian Health Services programs.

**Facility Type is an NCDB Data Item that refers to the general classification of the structural characteristics of the reporting facility. This classification is assigned by the Commission on Cancer Accreditation program.

***High Volume Facility status was assigned based on >90^th^ percentile by case volume.

Bolded p-values indicate statistical significance.

**Figure 2:**
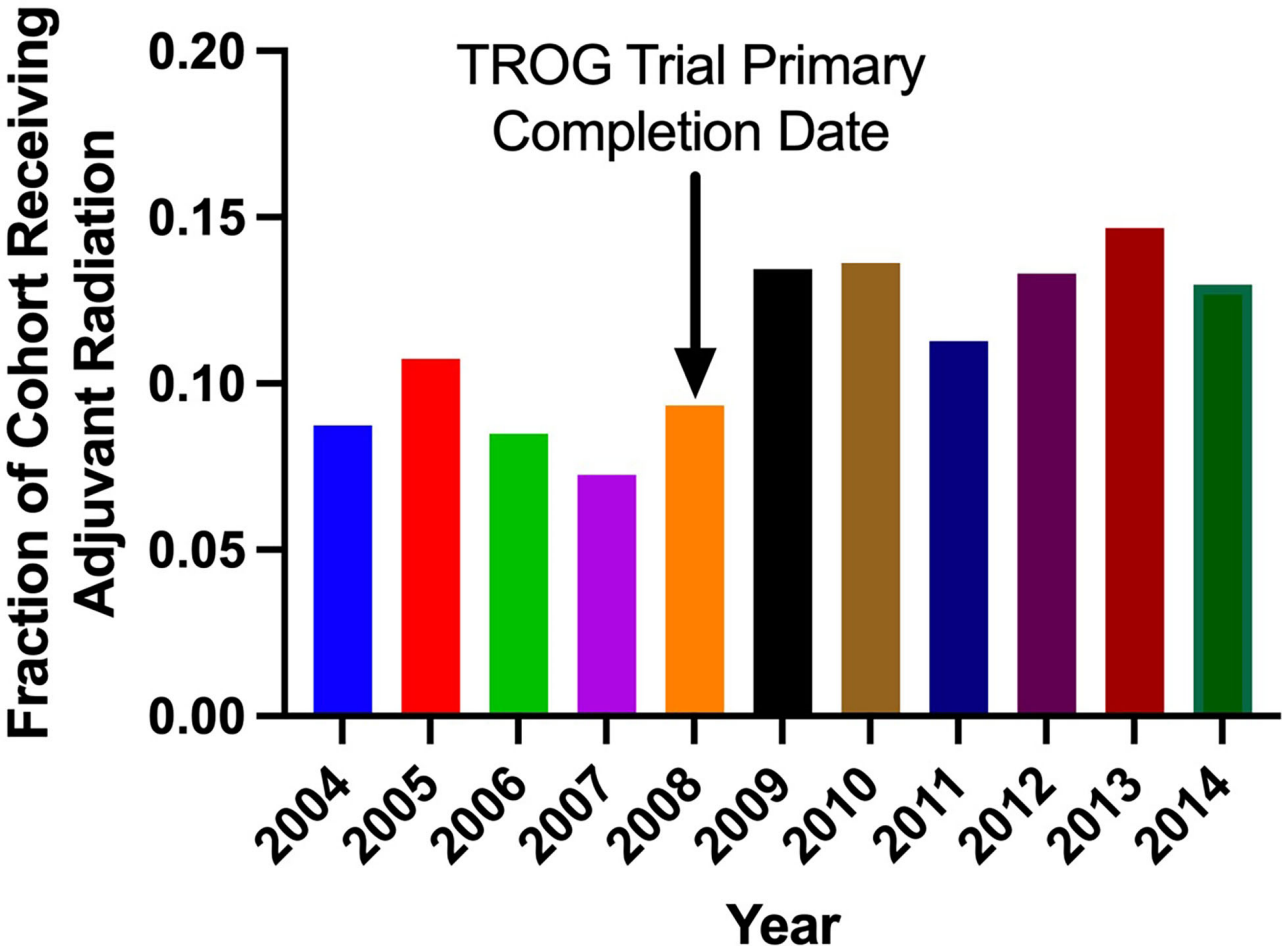
Fraction of cohort receiving adjuvant radiation in each year of diagnosis.

**Figure 3:**
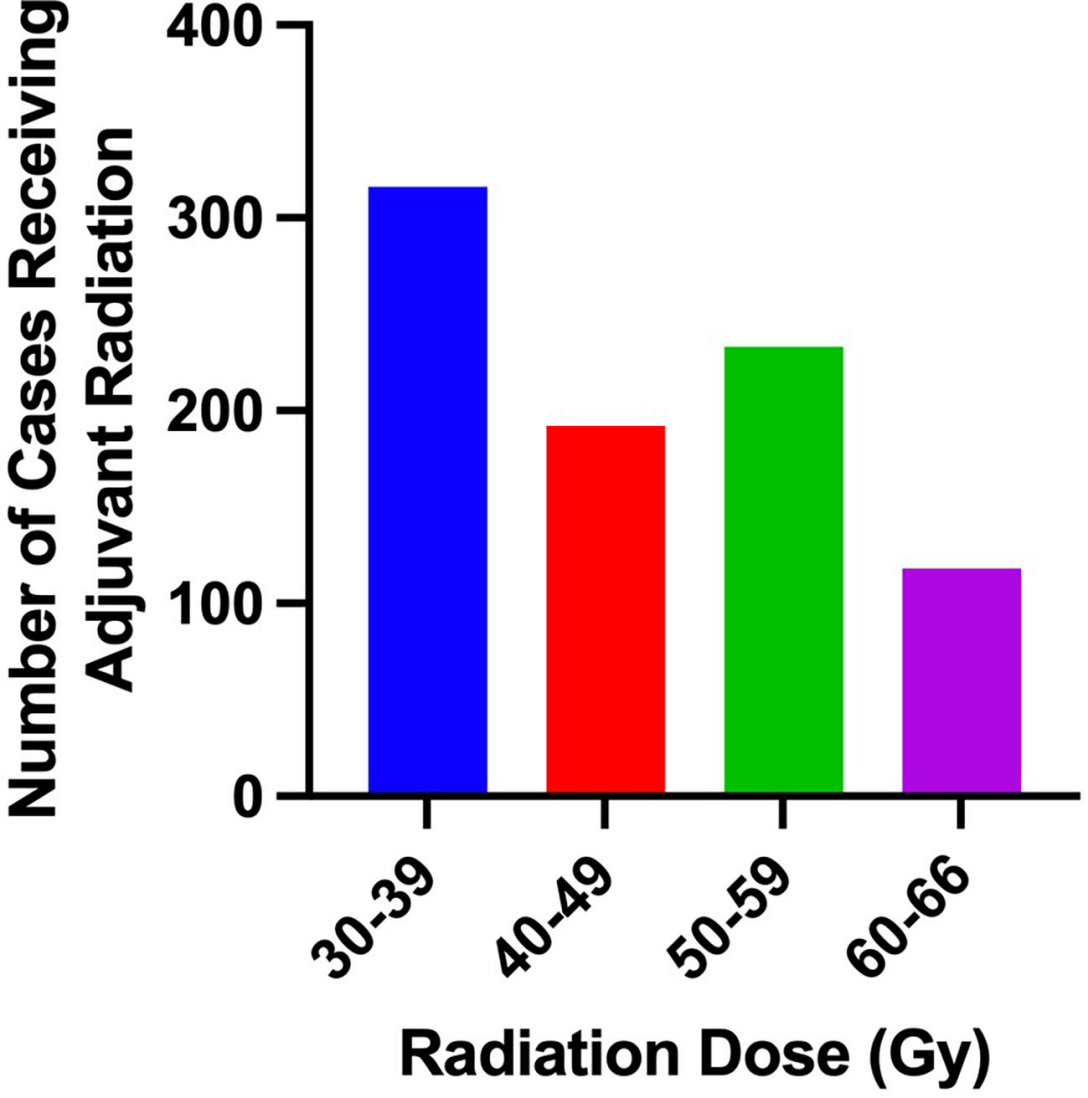
Number of cases receiving adjuvant radiation in each category of total radiation dosage.

**Figure 4:**
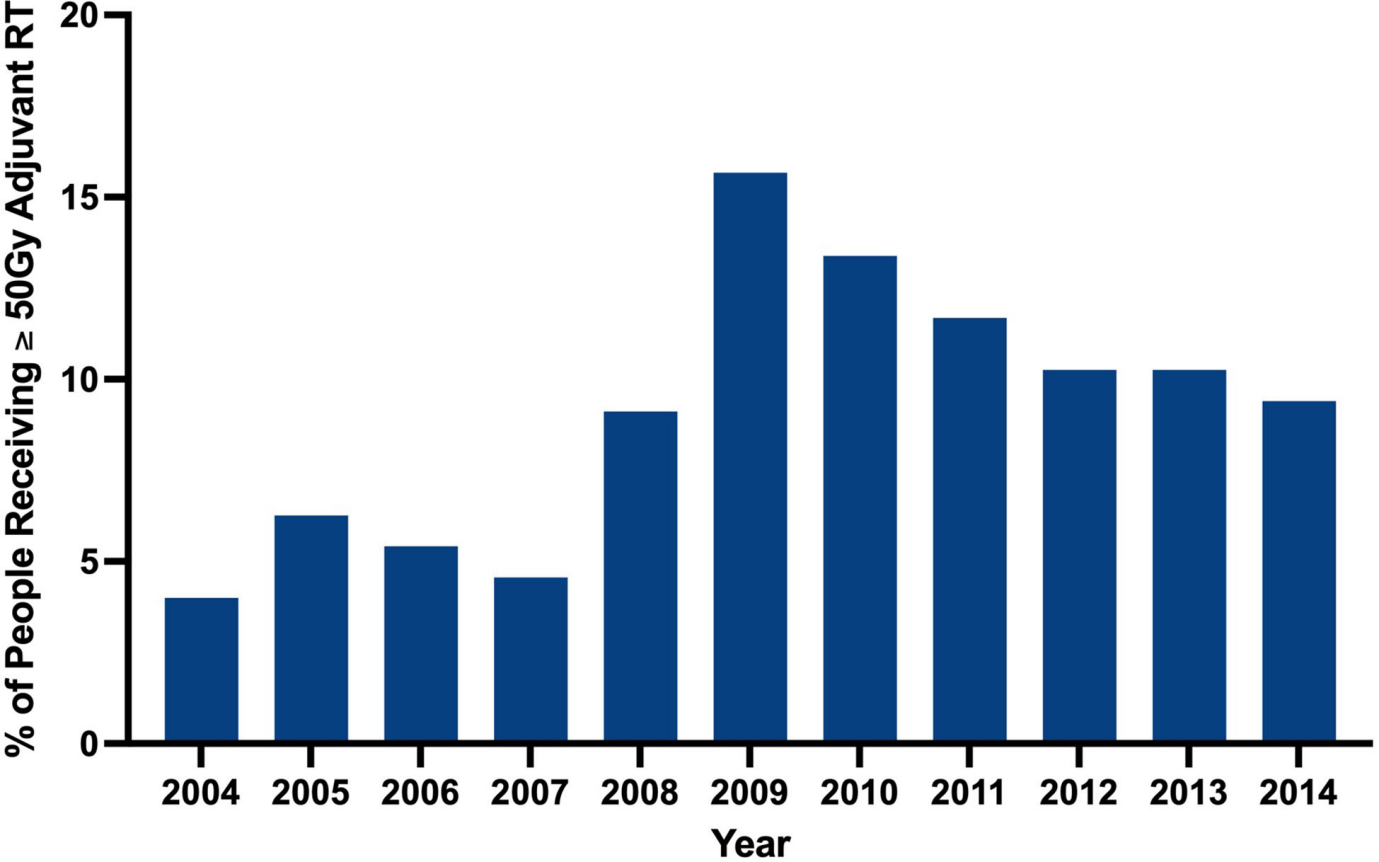
Percentage of cohort receiving **≥** 50 Gy of adjuvant radiation by year.

Clinical characteristics of patients that did and did not receive adjuvant RT are outlined in **Table 2**. The proportion of patients receiving adjuvant RT in each year are displayed in **Figure 2** and the dosages received by the patients are displayed in **Figure 3**. The proportion of patients receiving at least 50 Gy of radiation are displayed in **Figure 4**. The majority of patients had a Charlson-Deyo score of 0 (83.6%), less than 4 positive nodes (83.5%), and no residual tumor (90.8%).

**Table 2:** Clinical characteristics of patients in cohort.

Predictor Variable	No Adjuvant RT (Number of Patients [% of Patients Receiving No Adjuvant RT])	Adjuvant RT (Number of Patients [% of Patients Receiving Adjuvant RT])	Total (Number of Patients [% of All Patients])	P value
Charlson-Deyo Score				0.034
0	5,749 (83.96)	734 (80.57)	6,483 (83.57)	
1	901 (13.16)	145 (15.92)	1,046 (13.48)	
2	197 (2.88)	32 (3.51)	229 (2.95)	
Number of Positive Nodes				**<0.001**
<4	5,771 (86.76)	504 (58.47)	6,275 (83.51)	
4+	881 (13.24)	358 (41.53)	1,239 (16.49)	
Histology				**<0.001**
Malignant Melanoma, NOS	3,344 (48.84)	487 (53.46)	3,831 (49.38)	
Nodular Melanoma	1,723 (25.16)	224 (24.59)	1,947 (25.10)	
Lentigo Maligna Melanoma	50 (0.73)	17 (1.87)	67 (0.86)	
Superficial Spreading	1,302 (19.02)	109 (11.96)	1,411 (18.19)	
Acral Lentiginous Melanoma	181 (2.64)	20 (2.20)	201 (2.59)	
Desmoplastic Melanoma	53 (0.77)	19 (2.09)	72 (0.93)	
Spindle cell nevus, NOS	99 (1.45)	21 (2.31)	120 (1.55)	
Other	95 (1.39)	14 (1.54)	109 (1.41)	
Surgical Margin Status				**<0.001**
No residual tumor	6,274 (91.63)	768 (84.30)	7,042 (90.77)	
Residual tumor, NOS	193 (2.82)	36 (3.95)	229 (2.95)	
Microscopic residual tumor	155 (2.26)	51 (5.60)	206 (2.66)	
Macroscopic residual tumor	*	*	*	
Margins not evaluable	63 (0.92)	18 (1.98)	81 (1.044)	
Unknown or not applicable	150 (2.19)	32 (3.51)	182 (2.35)	
Immunotherapy				0.080
Did not receive immunotherapy	5,006 (73.11)	641 (70.36)	5,647 (72.79)	
Received immunotherapy	1,841 (26.89)	270 (29.64)	2,111 (27.21)	
Total	6,847	911	7,758	

*Values are censored to conceal values <10.

Bolded p-values indicate statistical significance.

On multivariable regression, multiple patient and hospital-related factors were associated with use of adjuvant RT. Patients who presented to the hospital in 2009-2014 as compared to 2004-2008 (odds ratio [OR] 1.61; 95% confidence interval [CI] 1.36-1.92), had 4 or more positive nodes (OR 4.30, 95% CI 3.67-5.04), had desmoplastic melanoma (OR 3.15, 95% CI 1.77-5.59), and had microscopic residual tumor (OR 2.11, 95% CI 1.46-3.04) were more likely to receive adjuvant RT. Female gender (OR 0.72, 95% CI 0.61-0.85), median income of at least $63,000 (OR 0.66, 95% CI 0.52-0.83), and a diagnosis of superficial spreading melanoma (OR 0.62, 95% CI 0.49-0.78) were negatively associated with receiving adjuvant RT (**Table 3**).

**Table 3:** Multivariable model for demographic, hospital-related factors, and disease characteristics associated with adjuvant radiation use.

Predictor Variable	OR (95% CI)	P Value
Year Category		
2004-2008	ref	
2009-2014	1.61 (1.36-1.92)	**<0.001**
Age Category		
18-19	ref	
50-64	1.30 (1.06-1.59)	0.011
65+	1.33 (1.08-1.63)	0.007
Sex		
Male	ref	
Female	0.72 (0.61-0.85)	**<0.001**
Race		
White	ref	
Black	1.97 (1.15-3.36)	0.014
Other	1.65 (1.04-2.60)	0.033
Median Income Quartiles 2008-2012		
< $38,000	ref	
$38,000 - $47,999	0.91 (0.71-1.15)	0.420
$48,000 - $62,999	0.87 (0.69-1.09)	0.231
$63,000 +	0.66 (0.52-0.83)	**<0.001**
High Volume Facility		
<90th% volume	ref	
>=90th% volume	0.86 (0.65-1.13)	0.274
Charlson-Deyo Score		
0	ref	
1	1.15 (0.93-1.41)	0.197
2	1.07 (0.70-1.63)	0.745
Number of Positive Nodes		
<4	ref	
>=4 nodes positive	4.30 (3.67-5.04)	**<0.001**
Histology		
Malignant Melanoma, NOS	ref	
Nodular Melanoma	0.84 (0.70-1.01)	0.062
Lentigo Maligna Melanoma	2.30 (1.26-4.22)	0.007
Superficial Spreading Melanoma	0.62 (0.49-0.78)	**<0.001**
Acral Lentiginous Melanoma	0.53 (0.32-0.89)	0.017
Desmoplastic Melanoma	3.15 (1.77-5.59)	**<0.001**
Spindle cell nevus, NOS	1.47 (0.87-2.49)	0.151
Other	0.90 (0.48-1.67)	0.732
Surgical Margin Status		
No Residual tumor	ref	
Residual tumor, NOS	1.15 (0.76-1.73)	0.510
Microscopic residual tumor	2.11 (1.46-3.04)	**<0.001**
Macroscopic residual tumor	2.96 (0.85-10.39)	0.090
Margins not evaluable	2.31 (1.26-4.23)	0.007
Unknown or not applicable	1.19 (0.75-1.90)	0.463
Immunotherapy		
Did not receive immunotherapy	ref	
Received immunotherapy	1.30 (1.10-1.54)	0.003

Bolded p-values indicate statistical significance.

## 4 Discussion

While surgery remains the mainstay of treatment for melanoma, for unresectable cases, high-risk surgical candidates, and those at high risk of toxicity to systemic adjuvant therapy, RT can be a powerful treatment modality. Although melanoma has been widely considered radio-resistant, with the past role of RT limited to palliative care, the applications for adjuvant RT in melanoma has been explored extensively in recent years. A phase II study TROG 96.06 found that the use of adjuvant RT in patients with melanoma involving lymph nodes was associated with a high rate of locoregional control (91%) (6). This study was followed by randomized study ANZMTG 01.02/TROG 02.01, which demonstrated that RT following lymphadenectomy for selected patients with node-positive melanoma reduces risk of locoregional recurrence (1). In this trial, the patients who derived the greatest benefit from treatment with adjuvant RT were those with pathological evidence of extracapsular extension (ECE) of nodal disease (1). Notably, we were unable to identify and thus analyze this subgroup of patients in our study due to database limitations. Despite evidence of benefit, there is no established consensus or clinical framework to guide the utilization of RT in melanoma. To this end, our study seeks to identify demographic, social, clinical, and hospital-specific factors associated with receipt of adjuvant RT and to examine the trends of adjuvant RT utilization over time.

### 4.1 Trend of adjuvant RT usage

Our analysis found that RT was utilized significantly more for stage III melanoma in more recent years (2004-2008 vs. 2009-2014; p<0.001). The wealth of recent studies in the literature that explore the use of RT in melanoma corroborate this observed trend. In a retrospective analysis of 1,675 patients with extracranial metastatic melanoma by Gabani et al., it was suggested that RT plays an increasing role in the management of metastatic melanoma in the era of immunotherapy (7). The rising use of RT in melanoma within the past decade may reflect the updated reappraisal of the value of adjuvant RT in melanoma. Our results suggest that higher RT dosages have been employed in more recent years despite lack of well established guidelines to dictate adjuvant RT dosing.

### 4.2 Demographic factors associated with adjuvant RT usage

Our analysis found that patients with stage III melanoma of higher income were significantly less likely to receive adjuvant RT (p<0.001). The discrepancy in disease severity upon presentation among different socioeconomic classes may serve as a possible explanation. In a retrospective review of 49,772 patients with cutaneous melanoma, Cormier et al. found that overall 5-year survival was significantly less in minorities than in their white counterparts, even after adjusting for age, sex, and region (8). Notably, minorities were significantly more likely to present with stage IV melanoma than were whites. These racial and socioeconomic disparities in severity of melanoma at presentation is corroborated by many other studies in the literature (9–11). Patients of lower income quartiles may be more likely to receive adjuvant RT possibly due to increased severity of disease that is not amenable to surgery upon presentation (12). Although our study did not find a statistically significant difference in receipt of RT with respect to insurance status (p=0.003), disparities in outcomes with respect to insurance status have been reported previously in the literature. In an epidemiological analysis of 26,958 melanoma patients by Kooistra et al., Medicaid patients were more likely to present with a late stage (9). Another retrospective study examining the association of health insurance with outcomes in 61,650 melanoma patients by Amini et al. found that patients with Medicaid or no insurance were more likely to present with increased tumor thickness and ulceration (12). A nationwide review of 15,941 metastatic melanoma patients conducted by Hague et al. revealed that African American race, Medicaid, and lower income status were associated with significantly decreased receipt of immunotherapy. In light of the social disparities in receipt of immunotherapy for melanoma (13), it is conceivable that disparities also exist in the receipt of RT.

### 4.3 Clinical factors associated with adjuvant RT usage

Increased disease burden, unresectable disease, and patients with high risk of locoregional spread are likely strong indicators for use of adjuvant RT. Consistent with this hypothesis, our study found that patients with at least 4 positive lymph nodes were more likely to receive adjuvant RT (p<0.001). The ANZMTG 01.02/TROG 02.01 trial stratified patients into categories of 4 or more involved lymph nodes and less than 4 involved lymph nodes, suggesting an important clinical distinction in the number of involved nodes (1). The trial additionally stratified patients based on ECE, finding that patients with ECE of nodal disease achieved the greatest benefit from treatment with adjuvant RT (1). Multiple studies in the literature have examined the impact of adjuvant RT on locoregional control in melanoma. In a retrospective review of 160 patients with cervical lymph node metastases from melanoma, Ballo et al. found that adjuvant RT use resulted in a 10-year regional control rate of 94% (3). In a study conducted by Owens et al. that examined the role of postoperative RT in mucosal melanoma of the head and neck, adjuvant RT versus surgery alone demonstrated a nonsignificant trend toward a lower rate of locoregional recurrence (p=0.13) (14).

Our analysis also found that patients with desmoplastic melanoma were also more likely to receive adjuvant RT than patients with other subtypes of melanoma (p<0.001). In line with this finding, in a retrospective study of 130 patients with nonmetastatic desmoplastic melanoma, Guadagnolo et al. found that adjuvant RT provided superior local control compared to surgery alone (15). This finding is reflected in the NCCN guideline to consider adjuvant RT in cases of high-risk desmoplastic melanoma (2).

Although a clear relationship between Charlson-Deyo score and receipt of RT was not identified in our study, the influence of increased comorbidities on treatment strategies in melanoma merits further investigation. It is possible that patients with increased disease burden and comorbidities may be deemed at higher risk for surgical complications, and thus, RT may be considered a more viable treatment option for such patients.

### 4.4 Potential disease benefits of adjuvant RT

Several studies have sought to examine the benefits of adjuvant RT for the treatment of melanoma. In a randomized phase III trial of 215 patients who underwent local treatment of one to three melanoma brain metastases assigned to whole brain radiation therapy or observation cohorts, no clinical benefit was observed (16). In a retrospective study of 56 patients with high-risk melanoma, Chang et al. found that adjuvant RT provided excellent locoregional control (4). Furthermore, they found that hypofractionation was equally effective as conventional fractionation. In a retrospective analysis of 200 melanoma patients with axillary metastases, Beadle et al. found that adjuvant RT to the axilla produced equivalent locoregional control when compared to adjuvant RT to both the axilla and supraclavicular fossa (5). The ANZMTG 01.02/TROG 02.01 trial was a randomized control trial that examined the use of adjuvant RT in melanoma patients who had undergone lymphadenectomy and were at high risk of recurrence (1). The trial found that adjuvant RT did not improve overall survival or relapse-free survival in the patient population analyzed, but did improve locoregional control.

The benefit of using RT in melanoma as adjuvant treatment has become particularly controversial in recent years given the promising results associated with the use of modern systemic therapies such as immune checkpoint and BRAF/MEK inhibitors, several of which have been approved for resected stage IIB, IIC, and III melanoma (17). Currently, systemic therapies approved as adjuvant therapy for melanoma include pembrolizumab, ipilimumab, and nivolumab as well as dabrafenib plus trametinib for patients with a BRAF V600 mutation (18). Our study revealed that patients who received immunotherapy were more likely to have received adjuvant RT, although this finding was not significant (p=0.003). Of note, since NCDB does not provide drug-specific data, we were unable to analyze the association between the specific type of immunotherapy and adjuvant RT.

Several studies have examined the combination of systemic therapy and RT in treating melanoma. In a retrospective cohort study examining 98 patients with melanoma lymph node metastases who underwent lymphadenectomy, patients receiving combined systemic and radiation therapies exhibited a lower 3-year cumulative incidence of lymph node basin recurrence relative to patients receiving only systemic therapy; however, the difference was not statistically significant (19). Additionally, no significant difference was noted in terms of in-transit/distant recurrences, disease-free survival, or melanoma-specific survival. In a retrospective study of 23 patients with mucosal melanoma, target lesion control rate at 1-year follow up was significantly higher in the cohort that received RT and pembrolizumab as compared to either treatment used alone (20). This suggests that immune checkpoint therapy may have a potential radiosensitizing effect on tumors. Several clinical studies support the existence of an interaction between immunotherapy and RT (21). In a phase I clinical trial, among seven patients with metastatic melanoma who were treated with stereotactic body radiation therapy followed by interleukin-2 (IL-2), 71% achieved a complete or partial response compared to 16% that was previously reported in association with IL-2 monotherapy (22, 23). Another retrospective study of melanoma patients with brain metastases revealed that 40% of those treated with ipilimumab prior to RT exhibited a partial response to RT whereas only 9% achieved a partial response among those who were not treated with ipilimumab (24). These results support the possibility that immunotherapy and RT act synergistically in the treatment of melanoma and can be used in combination as adjuvant therapy for stage III disease.

With the advent and increasing number of promising adjuvant systemic therapies for melanoma over the past decade, the role of adjuvant RT has potentially been superseded for most patients. However, adjuvant RT can be a powerful treatment modality particularly for patients who are not candidates for adjuvant systemic options. Patient characteristics that may favor RT over systemic therapies for adjuvant treatment include lack of a targetable BRAF mutation, high risk of toxicity to systemic therapies including those with a history of autoimmune disease, and/or patient preference. In these cases, consideration of adjuvant RT may be beneficial. Patients with significant ECE on pathology should also be considered for adjuvant RT with or without concurrent systemic therapy.

### 4.5 Limitations

The limitations of this study are inherent to retrospective database studies. The NCDB may contain coding and reporting biases and misclassified or incomplete data. Furthermore, relapse patterns could not be identified due to the lack of coding for local or distant recurrence, and we were unable to access detailed pathology information including the presence or absence of ECE. Nevertheless, this study elucidates trends of adjuvant RT utilization over time, and identifies demographic, social, clinical, and hospital-specific factors associated with receipt of adjuvant RT. Further investigation into the disease benefits of RT for the treatment of melanoma is warranted.

## Data availability statement

Publicly available datasets were analyzed in this study. This data can be found here: https://www.facs.org/quality-programs/cancer-programs/national-cancer-database/. The data used in the study are derived from a de-identified NCDB file. The American College of Surgeons and the Commission on Cancer have not verified and are not responsible for the analytic or statistical methodology employed, or the conclusions drawn from these data by the investigator.

## Ethics statement

Ethical review and approval was not required for the study on human participants in accordance with the local legislation and institutional requirements. Written informed consent for participation was not required for this study in accordance with the national legislation and the institutional requirements.

## Author contributions

AK, BY, and FM contributed to writing the manuscript. CZ, BY, and VL conducted the statistical analyses. DY, TT, JL, and YA provided oversight and guidance for this study. All authors contributed to the article and approved the submitted version.
